# A new species of *Brachiella* (Copepoda, Siphonostomatoida, Lernaeopodidae) from Peninsular Malaysia, with relegation of two genera *Charopinopsis* and *Eobrachiella* to junior synonyms of *Brachiella*

**DOI:** 10.1051/parasite/2020038

**Published:** 2020-05-28

**Authors:** Susumu Ohtsuka, Wojciech Piasecki, Norshida Ismail, Ahmad Syazni Kamarudin

**Affiliations:** 1 Takehara Station, Setouchi Field Science Centre, Graduate School of Integrated Sciences for Life, Hiroshima University 5-8-1 Minato-Machi Takehara Hiroshima 725-0024 Japan; 2 Institute of Marine and Environmental Sciences, University of Szczecin ul. Mickiewicza 16 70-383 Szczecin Poland; 3 School of Animal Science, Faculty of Bioresources and Food Industry, Universiti Sultan Zainal Abidin 22200 Besut Terengganu Malaysia

**Keywords:** *Brachiella*, *Charopinopsis*, *Eobrachiella*, Lernaeopodidae, Malaysia

## Abstract

Both sexes of *Brachiella malayensis* n. sp. are described on the basis of specimens found in the nostrils of narrow-barred Spanish mackerel *Scomberomorus commerson* (Lacepède) collected off Besut, Malaysia. The female of this species closely resembles those of *B. magna* Kabata, 1968 and *B. cybii* Pillai, Prabha et Balaraman, 1982 but is distinguishable mainly by the body size and the proportions of the cephalosome, posterior processes and caudal rami. While examining the male, we noticed a systematic inconsistency in some lernaeopodid genera. The genus *Brachiella* Cuvier, 1830, represented by its type-species *Brachiella thynni* Cuvier, 1830, and two monotypic genera *Charopinopsis* Yamaguti, 1963 and *Eobrachiella* Ho et Do, 1984, represented by *Charopinopsis quaternia* (Wilson, 1935) and *Eobrachiella elegans* (Richiardi, 1880), respectively, share distinct synapomorphies in the embracing (vs. pinching) elongate male maxilliped and the female trunk with a pair of long, cylindrical ventroposterior processes (in addition to a pair of modified caudal rami), both of which are involved in their unique reproductive strategy. The latter two genera are herewith relegated to junior synonyms of *Brachiella*.

## Abstract (fr)

Les deux sexes de *Brachiella malayensis* n. sp. sont décrits sur la base de spécimens trouvés dans les narines du Thazard rayé *Scomberomorus commerson* (Lacepède) collecté au large de Besut, en Malaisie. La femelle de cette espèce ressemble étroitement à celles de *B. magna* Kabata, 1968 et *B. cybii* Pillai, Prabha et Balaraman, 1982, mais se distingue principalement par la taille du corps et les proportions du céphalosome, les processus postérieurs et les rami caudaux. En examinant le mâle, nous avons remarqué une incohérence systématique dans certains genres de Lernaeopodidae. Le genre *Brachiella* Cuvier, 1830, représenté par son espèce-type *Brachiella thynni* Cuvier, 1830, et deux genres monotypiques *Charopinopsis* Yamaguti, 1963 et *Eobrachiella* Ho et Do, 1984, représentés par *Charopinopsis quaternia* (Wilson, 1935) et *Eobrachiella elegans* (Richiardi, 1880), respectivement, partagent des synapomorphies distinctes dans le maxillipède allongé (plutôt que pincé) et le tronc féminin avec une paire de longs processus cylindro-ventro-postérieurs (en plus d’une paire de rami caudaux modifiés), tous deux impliqués dans leur stratégie de reproduction unique. Les deux derniers genres sont ici relégués comme synonymes juniors de *Brachiella*.

## Introduction

Females of the parasitic copepod family Lernaeopodidae are unique in their highly modified bodies, possession of an attachment organ called the bulla joining both maxillae in females, and parasitism on gills, mouths, nostrils, eyes, fins, and skins of both marine and freshwater fishes [25, 43, 64]. Dwarf males adhere to bodies of the giant females with their maxillae and maxillipeds [25]. Some lernaeopodids such as *Clavella* Oken, 1815 and *Salmincola* Wilson C.B., 1915 have caused negative impacts on marine and freshwater farmed fishes, respectively [20, 38, 44].

During our taxonomic survey on parasitic copepods of marine fishes in Malaysia, we found specimens of copepods resembling *Brachiella magna* Kabata, 1968 but also differing from it. The copepods were collected from the nostrils of narrow-barred Spanish mackerel *Scomberomorus commerson* (Lacepède). Both sexes of this copepod are described herein. This is the first Malaysian record of the occurrence of a species representing the genus *Brachiella*.

The largest and most diversified group of the family Lernaeopodidae is the “*Brachiella*-group” deriving its name from the genus *Brachiella* Cuvier, 1830. The group is “most highly advanced and apparently very actively speciating, it abounds in species, the large number and the great morphological variety of which add to the difficulties of a sensible taxonomic arrangement” [25]. In fact, the taxonomy of the *Brachiella*-group has been confusing for decades. It was further complicated by ambiguous definitions and broad interpretation by Wilson [64]. The first comprehensive approach to this large informal “taxon” was by Kabata [25] who not only challenged Wilson’s [64] concepts, but also established the genus *Neobrachiella* Kabata, 1979 to accommodate species differing from the *Brachiella* type species and sharing some important taxonomic features. These species were previously assigned to *Brachiella*, *Parabrachiella* Wilson, C.B., 1915, *Probrachiella* Wilson C.B., 1915, *Epibrachiella* Wilson C.B., 1915, *Branchiellina* Pearse, 1952, *Lernaeopoda* von Nordmann, 1832 and *Isobranchia* Heegaard, 1947. The new generic name persisted unchallenged until 2004 when Boxshall and Halsey [6] noticed that the name *Parabrachiella* should have priority over the name *Neobrachiella*. As a consequence, many species accommodated in *Neobrachiella* had to be transferred to *Parabrachiella* [31, 46]. Kabata [25] not only re-ordered the family Lernaeopodidae, including the *Brachiella*-group, based on female morphology, but also focused his attention on the morphology of the male. He divided lernaeopodid males, based on their gross morphology, into three types, A, B and C. The most abundant in genera was type A accommodating, among others, the *Brachiella*-group and freshwater lernaeopodids. Kabata [25] noticed that males of *Brachiella* and *Neobrachiella* differ in the structure of their maxillae (“second maxillae”) and maxillipeds but he failed to draw conclusions.

Yamaguti [66] and Ho and Do [16] established monotypic genera, *Charopinopsis* and *Eobrachiella*, accommodating *Charopinus quaternia* Wilson, 1935 [65] and *Brachiella elegans* Richiardi, 1880 [51], respectively. However, close and critical examination of these three genera reveal common morphological characters, which have not hitherto been used for discriminating higher taxa of lernaeopodid copepods. Ho and Do [16] have put forward a hypothesis concerning the reproductive and evolutionary trend of a lernaeopodid lineage. Their hypothesis provides a framework for our analysis on the morphological adaptations of the male and to propose a fundamental change in discriminating lernaeopodid copepods of the *Brachiella*-group.

## Materials and methods

A host fish head was purchased at a market in Besut, Terengganu, Malaysia (5.8312° N, 102.5619° E) on 15 October 2019 by the third and fourth authors. Since only the head was available for parasitological examination, the identity of the fish host species was determined using DNA barcoding based on a fragment of the cytochrome c oxidase subunit I (COI) gene in the mitochondrial genome. Approximately 1 cm^2^ of tissue of the fish species was removed with scissors and preserved in a sterile 1.5 mL tube containing 95% ethanol. The total genomic DNA of the fish was isolated using a Favorgen DNA extraction Kit (Favorgen Biotech Corp., Ping-Tung 908, Taiwan). The partial COI gene of mitochondrial DNA was amplified using PCR with the universal primers of COI-Fish2 F (5′–TCGACTAATCATAAAGATATCGGCAC–3′) and COI-Fish2 R (5′–ACTTCAGGGTGACCGAAGAATCAGAA–3′) [62]. The PCR was carried out in a 25 μL reaction volume containing 18.2 μL sterile distilled water, 2.5 μL Taq buffer, 2.0 μL dNTP Mix (2.5 mM), 0.5 μL of each primer (10 μM), 0.3 μL of 5 unit μL^−1^ Taq polymerase (TaKaRa) and 1 μL template DNA (50 ng μL^−1^) on a thermal cycler PCR machine Veriti 96 Well Thermal Cycler (Applied Biosystems, California, USA). The thermal cycling conditions started with initial denaturation at 95 °C for 5 min; 35 cycles including denaturation at 95 °C for 30 s, annealing at 50 °C for 30 s and elongation at 72 °C for 10 min; followed by a final extension for 10 min at 72 °C and then the PCR product was maintained at 4 °C. Sequencing was carried out using a BigDye Terminator v3.1 Cycle Sequencing kit (Applied Biosystems), following the manufacturer’s instructions, performed on an ABI Prism 3730XL Genetic Analyzer (Applied Biosystems). Two COI were aligned and edited using the ClustalW multiple sequence alignment program in MEGA 7 [30]. DnaSP software was used to determine the variable sites among the sequence [34]. To discover the origin of the fish species, the sequenced haplotype was queried using the Basic Local Alignment Search Tool (BLAST) against the National Center for Biotechnology (NCBI) nucleotide database and the BOLD identification engine [50]. A top species match was identified with sequence similarity of at least > 90% to avoid false positives.

The copepods were recovered from the nostrils of the fish host and were preserved in 70% ethanol for further examination. The copepods were observed in lactophenol on Humes and Gooding’s [17] slides, and illustrated with the aid of a drawing tube attached to a microscope (Olympus BX53). The type specimens are deposited at the South China Sea Repository and Reference Center, University Malaysia Terengganu, Malaysia (UMTCrus 1099 and UMTCrus 1100). The terminology used follows Huys and Boxshall [18] and Kabata [25].

## Results

### Host identification

After removal of low quality sequences at the 5′ and 3′ ends, a 629 bp DNA barcode was obtained in FASTA format. The sequence was deposited in GenBank with the accession number MT423724. The host sequence showed 100% similarity with sequences of the same species respectively in GenBank (MG220579) and in BOLD identification engine [50], including sequences of the same species collected off the coast of China [5]. This confirms the identification of the host species.

### Taxonomy

Class: Copepoda Milne Edwards, 1840

Order: Siphonostomatoida Burmeister, 1835

Family: Lernaeopodidae Milne Edwards, 1840

Genus: *Brachiella* Cuvier, 1830

*Synonyms*. *Charopinus* Krøyer, 1863 [48]; *Thynnicola* Miculicich, 1904 [57]; *Charopinopsis* Yamaguti, 1963 (new synonymy); *Eobrachiella* Ho et Do, 1984 (new synonymy).

*Diagnosis* (*amended*). Female. Body comprising cylindrical cephalothorax and trunk arranged in line; head slightly enlarged with distinct dorsal shield. Maxillae not medially fused and with bulla attached to host. Maxillipeds substantially displaced in front of maxillae. Trunk cylindrical or more or less dorsoventrally flattened, with pair of dorsoposterior processes (= modified caudal rami) and pair of ventroposterior processes. Gonopores and copulatory pores located ventrolaterally, anterior to caudal rami. Modified caudal rami almost equal to or longer than posterior processes. Antennule 3- or 4-segmented; terminal segment with typical lernaeopodid setation. Antenna heavily sclerotized, with 1- or 2-segmented endopod at angle or parallel to exopod with typical lernaeopodid setation at tip. Mandible with 2 or 3 secondary teeth. Maxillule bilobed, with 2 setae on palp and 3 setae on endite. Maxilliped subchelate; subchela with terminal claw with or without auxiliary spine. Egg sac multiseriate, long.

Male. Body consisting of large cephalothorax with distinct dorsal shield and variably developed trunk. Posterior processes of trunk present or not. Paired gonopores located anterior to caudal rami. Caudal rami unarmed or with rudimentary elements, short or long. Antennule 4-segmented, with typical lernaeopodid setation. Antenna biramous; exopod unilobed, with 2 subterminal elements; endopod 2-segmented, with typical lernaeopodid armament. Mandible and maxillule similar to those of female. Both right and left maxillae linked basally by cuticular tympanum; subchelate, 2-segmented, proximal segment stout, without inner projection at distal corner; distal segment strongly curved inward. Maxilliped embracing (not pinching), elongate, and slender, 2-segmented; corpus with 3 denticulate processes, middle of which bearing element; subchela smoothly curved inward, with denticulate process subterminally and 1 spine or process and 1 small element terminally. Male attached to caudal ramus of female.

*Type*. *Brachiella thynni* Cuvier, 1830 [11]

*Other species*. *Brachiella elegans* Richiardi, 1880; *Brachiella quaternia* Wilson, 1935; *Brachiella seriolae* Yamaguti et Yamasu, 1960; *Brachiella magna* Kabata, 1968; *Brachiella cybii* Pillai, Prabha et Balaraman, 1982. (Note that the paper nominally listed as 1977 was published in 1982.)

*Remarks*. Historically, the taxonomy of Lernaeopodidae was based on female morphology. Also, Wilson [64] depended heavily on female morphology without noticing differences between males of *Brachiella* and other related genera. Kabata [25] pointed out that the genus *Brachiella* had accommodated miscellaneous species, and that only the type species *B. thynni* and *B. magna* could be adequately assigned to it. This statement was later repeated by Pillai [48]. Boxshall and Halsey [6] listed lernaeopodid genera with respective number of species. However, they mentioned “2” species of *Brachiella*, without listing the actual species names. In addition, Pillai et al. [49] suggested that *B. elegans*, *B. seriolae* and *B. gracilis* Wilson, 1908 [63] should be re-examined to clearly distinguish *Brachiella* from other lernaeopodid genera. The study is intended to follow suggestions of some of our predecessors.

Within the last 25 years, many species of the *Brachiella*-group have been re-examined and their generic identity confirmed or changed. One of the major obstacles in the classification is the lack of male descriptions in many species.

We decided to include *Brachiella cybii* in our list of valid species of the newly defined *Brachiella*, even though the male of *B. cybii* has not been described. This is because the latter species, recovered from the nasal cavity of *Acanthocybium solandri* (Cuvier), is very similar to *B. magna*, collected from the gills of *S. commerson*, but it differs in the body proportions (see Table 1).

**Table 1:** A comparison of morphometric and meristic features of females of *Brachiella* species.

Parameter	Species						
	*B. malayensis* n. sp.	*B. magna*	*B. cybii*	*B. thynni*	*B. elegans*	*B. quaternia*	*B. seriolae*
Cephalosome length (CL) [mm]	3.00	6.57	7.47	6	2.78	1.67	2.75
Cephalosome width [mm]	1.35	1.26	2.20	1	1.22	1.17	1.40
Trunk length (TL) [mm]	3.39	7.94	12.52	6	4.78	4.22	4.83
Trunk width [mm]	1.09	2.07	2.00	3	1.44	1.33	2.58
Total length [mm]	6.39	15.0	20.0	12	7.56	5.89	7.58
TL–CL ratio	1.13	1.33	1.68	1.00	1.72	2.53	1.76
Dorsoposterior process length (DPL)	1.48	4.20	4.51	10.00	1.00	0.78	1.80
Ventroposterior process length (VRL)	1.35	4.23	3.74	7.50	3.33 L, 4.33 R	2.89	5.20
DPL–TL ratio	0.44	0.53	0.23	1.38	0.21	0.18	0.37
VRL–TL ratio	0.44	0.53	0.19	1.04	0.70 L, 0.91 R	0.68	1.08
Anal tubercle	Present	Present	–	Present	Present	Absent	Present
Antennulary segmentation	4	4	4	4	4	3 or 4*	4
No. of elements on antennary endopod tip	3	–	–	3	3	3	2
Anteriormost seta on maxillulary palp	Developed	Developed	–	Developed	Reduced	Reduced	Reduced
Maxillae length	< CL?	= CL	>> CL	< CL	< CL	< CL	< CL
Patch of denticles on maxillipedal subchela	Absent	Present	–	Present	Present	Present	Present
Maxillipedal claw with auxiliary spine	No	No	–	No	Yes	Yes	Yes

Measurement of *B. magna* based on Kabata [22]; measurements for *B. cybii* were based on direct measurement form Pillai et al.’s [49] figure; measurements for *B. thynni* were based on Wilson [64]; measurements for *B. elegans*, *B. quaterina*, and *B. seriolae* were based on figures or measurement of Ho and Do [16];

* from Kabata [21]; –: no data provided; L = left. R = right.

In addition to the species mentioned above that we have decided to include in the newly defined genus *Brachiella*, there are also seven hitherto unchallenged *Brachiella* species that require alternative taxonomic assignment: *B. sciaenophila* (Heller, 1865); *B. ovalis* van Beneden, 1871; *B. fasiculata* (Leidy, 1889); *B. gracilis*; *B. lageniformis* Szidat, 1955; and *B. parva* Nuñes-Ruivo, 1957. *Brachiella ovalis* (“*Anchorella ovalis*”) is *Clavellisa emarginata* (Krøyer, 1837) but “*Anchorella ovalis* Krøyer” of van Beneden [59] and “*Brachiella ovalis* (Krøyer)” of Scott and Scott [53] both represent *Parabrachiella bispinosa* (von Nordmann, 1832) [25]. *Brachiella sciaenophila* (“*Anchorella sciaenophila*”) [15] and *B. fasiculata* (“*Anchorella fasiculata*”) [32] may be moved to *Parabrachiella* in consideration of the medially fused maxillae of the female. *Brachiella neglecta* Richiardi, 1880 is a nomen nudum (Article 12, ICZN [19]). “*Brachiella* (*neglecta* Richiardi)?” of Brian [7] should be *Parabrachiella chevreuxii* (Beneden, 1891) [25]. Females of *B. gracilis* have fused maxillae, and its dwarf males cling to the cephalosome of the mate [63]. As such, they clearly represent the “pinching” attachment strategy (vs. embracing strategy of the newly defined *Brachiella*). Therefore, these should not be included in *Brachiella*.

The original description of *B. lageniformis* does not provide enough detail about the male [58]. A re-description of both sexes of *B. lageniformis* provided by Kabata [24] showed that the species has no synapomorphy of *Brachiella* redefined in our paper. It should be newly transferred to *Parabrachiella* (see below in Section “Discussion”), because it was not listed in Piasecki et al. [46] nor Lebepe and Dippenaar [31].

*Brachiella parva* has three pairs of short and thick posterior processes in the trunk [40]. Although the male was also described and illustrated by Nuñes-Ruivo [40], the maxillipeds were not described in detail. Judging from the figure of the whole body (Fig. 2i, p. 98), the male maxilliped seems to be short, unlike that of *B. thynni*.

Another species, that may be erroneously synonymised with *B. magna* is *Epibrachiella magna* Song et Chen, 1976 [55]. Its female, however, bears single maxillary processes and three pairs of posterior processes (one pair corresponding to caudal rami) (see Piasecki et al. [46]), and the male does not have long maxillipeds such as seen in *B. thynni* (see Fig. 8L in [53]). This species is distinctly different from *B. magna*, and should be moved to the genus *Thysanote* Krøyer, 1863 as *Thysanote magna* (Song et Chen, 1976) (new combination).

The genus *Charopinopsis* was established to accommodate a single species *Charopinus quaternius* (Wilson, 1935) by Yamaguti [66], in which only the generic diagnosis was provided without any comparison between the newly established genus and its related genus *Charopinus*. The reason behind Wilson’s [65] suggestion about the alleged relation of this copepod with the *Charopinus*-branch was his false belief about the lack of bulla in *C. quaternius*. This false assumption was sustained by Yamaguti [66]. Later, Kabata [25] recognized the validity of the monotypic genus in consideration of the evolution of the family Lernaeopodidae, and pointed out its phylogenetic position on the *Brachiella* lineage. Kabata [21] and Ho and Do [16] carefully redescribed *Charopinopsis quaternia*. Only Ho and Do [16], however, redescribed the male. Kabata [21] proved that the species indeed has a small bulla and listed *Brachiella coryphaenae* Pearse, 1952 [42] as a junior synonym of *C. quaternia*.

The genus *Eobrachiella* was erected by Ho and Do [16] to accommodate *B. elegans*. The main differences between *Eobrachiella* and *Charopinopsis* were the presence or absence of an anal tubercle in the female and the shape of the caudal rami in the male [16].

However, these three genera share unique maxillipeds of the males, which can be regarded as a distinct synapomorphy. According to Ho and Do [16], the elongate maxillipeds of the males are most likely specialized to embrace the cylindrical ventroposterior process of the females, rather than pinching to avoid detachment from the females parasitic on fast-swimming pelagic fish. The male maxilliped is evidently characterized by three denticulated pads along the inner margin of the corpus, the middle of which has a setule, and an inner, terminal, denticulated pad of the subchela. Such an embracing maxilliped is found in the following six species, including the new species described herein: *B. thynni*; *B. elegans*; *B. quaternia*; *B. seriolae*; *B. magna*; and *B. malayensis* n. sp. described below. *Brachiella seriolae* was described by Yamaguti and Yamasu [67], but later, was relegated to a subspecies of *Eobrachiella elegans* by Ho and Do [16]. However, we think in consideration of the morphology of both sexes that these are two separate species. In females, the proportion of the cephalosome to the trunk and the fine structure of the antenna are different between these species (Table 1). In males, the general body shape and the morphology of the maxillipeds differ remarkably between these species (Table 2). In addition, these parasitized two different host species of the genus *Seriola* inhabiting the distant localities: *B. elegans* in the Atlantic and *B. seriolae* in the Pacific (Table 1).

**Table 2:** A comparison of morphometric and meristic features of males of *Brachiella* species.

Parameter	Species				
	*B. malayensis* n. sp.	*B. thynni*	*B. elegans*	*B. quaternia*	*B. seriolae*
Angle between cephalosome and trunk	Right angle	In line	In line	Right angle	In line
Cephalosome length (CL) [mm]	0.56	0.89	0.60	0.57?	0.51
Trunk length (TL) [mm]	1.03	1.14	0.67	Highly reduced	0.51
Total length [mm]	1.59	2.03	1.27	0.57	1.02
TL–CL ratio	1.84	1.28	1.12	–	1.00
Ventroposterior processes	Present	Present	Absent?	Absent?	Absent?
Dorsoventral processes (caudal rami)	Fusiform	Fusiform	Fusiform	Elongate, cylindrical	Fusiform
Antennulary segmentation	4	4	4	?	4
No. of elements on antennary endopod tip	3	3	4	4	3
Anteriormost seta on maxillulary palp	Developed	Developed	Reduced	Reduced	Reduced
Proximal process of maxillipedal corpus	Large	Large	Small	Small	Large
Fraction of imMXPs denticulate patch to segment size	> 1/2	> 1/2	ca. 1/6	ca. 1/4	ca. 1/5
Attachment of male to female ventroposterior processes	?	Yes	Yes	Yes	Yes
Reference	Present study	[25, 54, 65]	[16, 65]	[16, 21, 66],	[16]

The measurements for *B. thynni* were based on Wilson [64]; measurements for *B. elegans*, *B. quaternia*, and *B. seriolae* were based on figures or measurement of Ho and Do [16]; imMXPs = inner margin of maxilliped subchela.

Females of these six species share the following features: (1) the female body plan belongs to “Type A” sensu Kabata [25], meaning that the cephalosome and trunk are positioned in line (presumably in a relatively primitive condition); (2) the cephalosome is cylindrical (advanced); (3) pairs of ventro- and dorso-posterior processes of the trunk are well developed, elongate (advanced); (4) the antennae are not prehensile (advanced); (5) the mandible bears primary and secondary teeth (advanced); (6) the maxillae are not medially fused (primitive). Since the six above-mentioned species are well defined by the synapomorphic states of the male maxillipeds and female bodies, we propose herein that *Charopinopsis* and *Eobrachiella* are relegated to junior synonyms of *Brachiella* (new synonymy).

A closely related genus *Parabrachiella* is now a very specious taxon but unfortunately, in many species only females are known. Since a revision of the genus is beyond the scope of this paper, it should be briefly commented on the basis of the male maxillipeds of the type species as follows: male maxilliped subchelate of pinching type, similar in structure and function to maxilla; corpus unarmed; subchela short, with claw positioned at right angle to shaft; tip of claw fitting depression of medial triangular process of corpus during its closure (cf. Fig. 8, Plate LXIII in [53]). Piasecki et al. [46] transferred *B. elegans* and *B. seriolae*, both of which were assigned to *Eobrachiella* by Ho and Do [16], to the genus *Parabrachiella* without any comment on *Eobrachiella*.

The homology of ventro- and dorso-posterior processes of the females of *Brachiella* can be traced in consideration of the counterparts of the males of *B. thynii* and *B. malayensis* n. sp. The dorso-posterior processes represent caudal rami (see Fig. 2C).

### *Brachiella malayensis* Ohtsuka, Piasecki, Norshida et Ahmad-Syazni n. sp. (Figs. 1 and 2)

urn:lsid:zoobank.org:act:06D0D2E3-6A51-4628-8B27-3A72C97A45D6

**Figure 1:**
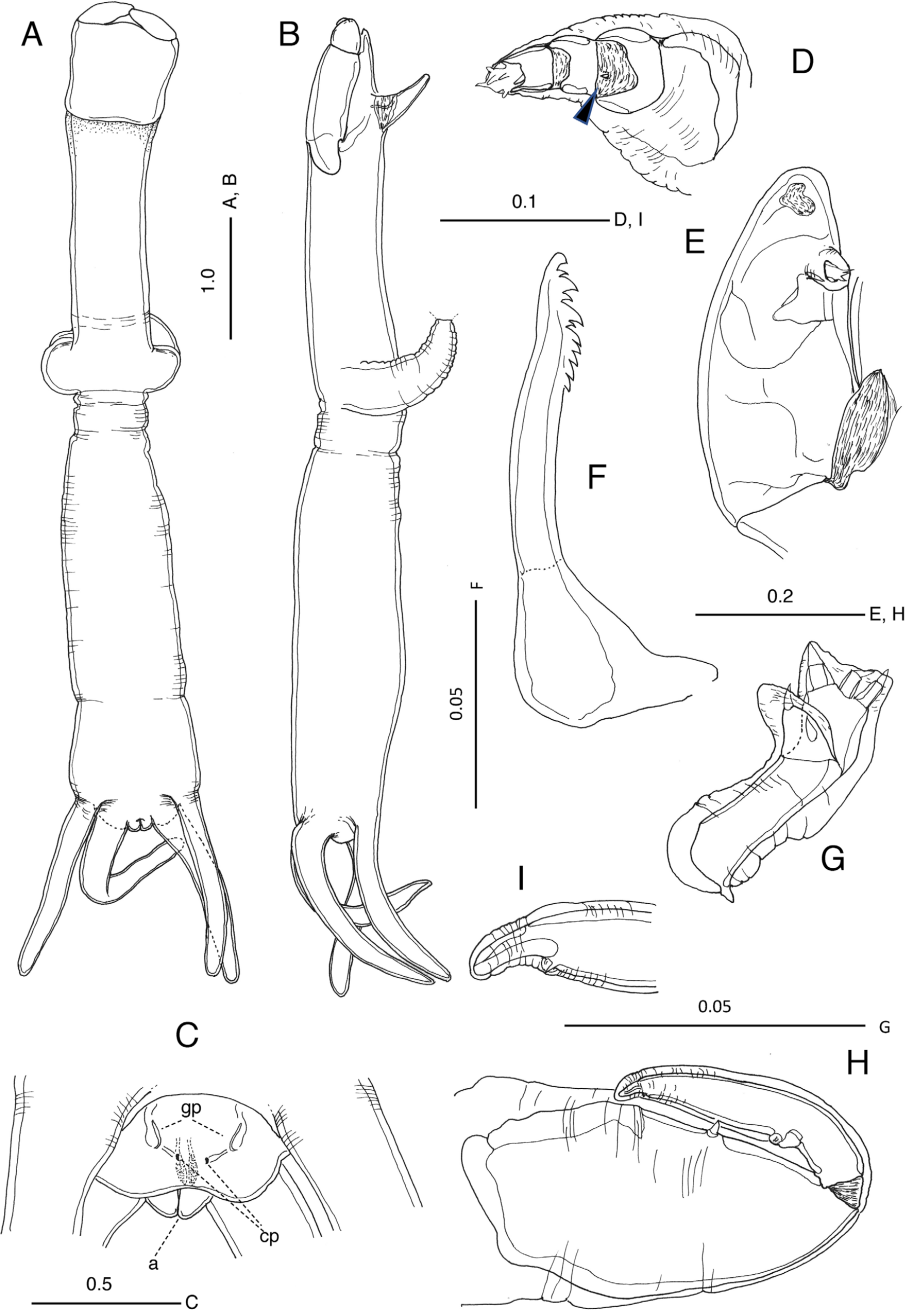
*Brachiella malayensis* n. sp., female, based on a specimen found from *Scomberomorus commerson* collected off Besut, Terengganu, Malaysia. (A) Habitus, dorsal view; (B) habitus, lateral view; (C) genital area, ventral view, (a) anus, (gp) gonopore, (cp) copulatory pore; (D) antennule, reduced element arrowed; (E) antenna; (F) mandible; (G) maxillule; (H) maxilliped; (I) tip of maxilliped. Scales in mm.

**Figure 2:**
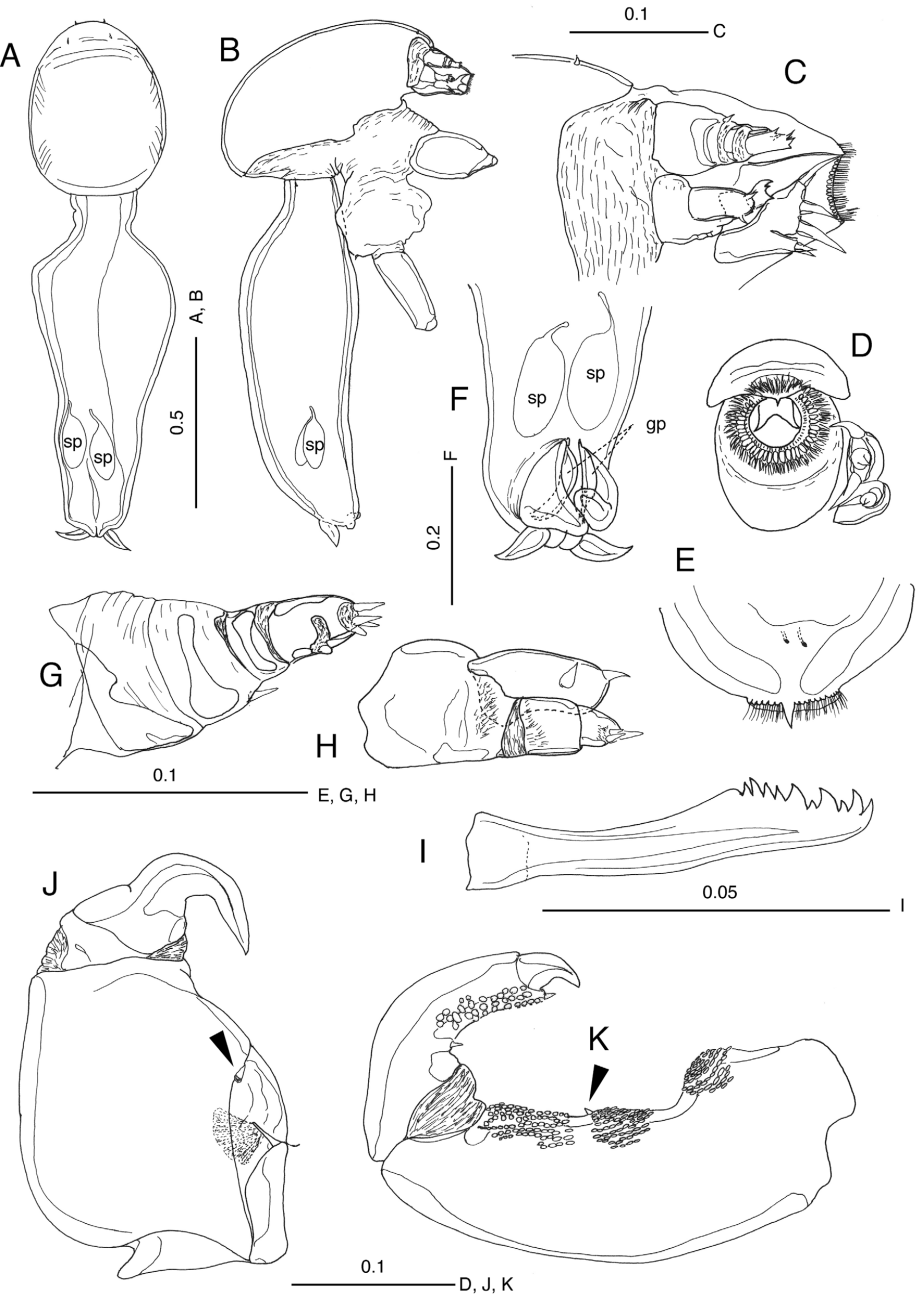
*Brachiella malayensis* n. sp., male, based on a specimen found from *Scomberomorus commerson* collected off Besut, Terengganu, Malaysia. (A) Habitus, dorsal view; (B) habitus, lateral view; (C) mouth area, lateral view; (D) oral cone and maxillule, ventral view; (E) labrum, ventral view; (F) genital area, ventral view; (G) antennule; (H) antenna; (I) mandible, (J) maxilla, short element arrowed; (K) maxilliped, short element arrowed. Scales in mm.

*Type-specimens*. Holotype, adult female, partly dissected and mounted on 1 slide, body in vial (UMTCrus 1099). Allotype, adult male, partly dissected and mounted on 1 slide, body in vial (UMTCrus 1100). Both specimens were found in the nostrils of *Scomberomorus commerson* purchased at a fish market in Besut, Terengganu, Malaysia on 15 October 2019.

*Type-locality*: Off Besut, Terengganu, Malaysia.

*Etymology*. The new specific name is derived from the type locality.

*Description*. Female. Body (Figs. 1A and 1B) consisting of cylindrical cephalosome (3.00 mm in length, 1.35 mm in maximum width at point of maxillae) and trunk (3.39 mm in length, 1.09 mm in maximum width near base of dorsal processes) in line, 6.39 mm measured from anterior tip of cephalosome to posterior end of trunk. Cephalic shield (0.80 mm in length, 0.89 mm in maximum width) heavily sclerotized, truncate along anterior margin. Maxillae (Fig. 1B, distal parts broken during processing) forming bulla not medially fused, located at ca. anterior 0.47 of total length. Trunk (Fig. 1B) more or less dorsoventrally flattened around posterior ends; paired ventroposterior processes almost equal in length to dorsoposterior processes (= caudal rami). Gonopores (“gp” in Fig. 1C) and copulatory pores (“cp” in Fig. 1C) located ventrolaterally, anterior to base of ventroposterior processes. Mucus remaining on copulatory pores (not illustrated), suggesting post-mating. Anal tubercle (Fig. 1C) located posteriorly, located between caudal rami; anus opening (“a “in Fig. 1C).

Antennule (Fig. 1D) 4-segmented with segments distinctly tapering distally; Second segment with reduced minute seta (whip), third segment unarmed, terminal segment equipped with at least six elements (of unequal size) at tip. Antenna (Fig. 1E) biramous, heavily sclerotized; endopod rudimentary, 1-segmented, positioned at right angle to exopod, having three short elements at tip; exopod lobate, apparently unarmed. Mandible (Fig. 1F) with three primary, three secondary and five basal teeth. Maxillule (Fig. 1G) bilobed, with two short setae on palp and three short setae on endite. Maxilla partly broken during removal from host, lacking terminal parts. Maxilliped (Figs. 1H and 1I) with stout corpus with short inner seta midway; subchela with short element at 1/3 length of inner margin, 1 large terminal claw and 1 minute auxiliary spine.

Male. Body (Figs. 2A and 2B) consisting of semioval cephalosome (cephalic shield: 0.70 mm in length, 0.49 mm in maximum width) and longer fusiform trunk (1.03 mm in length, 0.42 mm in maximum width); trunk joined at posteroventral side of cephalosome at right angle, constricted anteriorly, abruptly expanded laterally, and more or less asymmetrically tapering distally in dorsal view (Fig. 2A). Posterior processes of trunk (Fig. 2F) short, just anterior to and covering gonopores. Paired gonopores (Fig. 2F) located anterior to caudal rami. Caudal rami unarmed, shorter than posterior processes. Paired spermatophores (Figs. 2A, 2B, 2F) seen inside posterior part of trunk, oval in ca. 0.15 mm × 0.07 mm in size. Antennule (Fig. 2G) four-segmented, second segment with short element (whip) anteriorly, fourth segment having six elements at tip. Antenna (Fig. 2H) biramous; exopod lobate, slightly shorter than endopod, with one subterminal and one terminal element; exopod two-segmented, proximal segment with patch of fine spinules, distal segment having three unequal elements at tip. Mandible (Fig. 2I) with three primary, two secondary and five basal teeth. Oral cone (Figs. 2C, 2D, 2E) situated anteriorly; mouth fringed by membrane enforced with fine setules. Maxillule (Figs. 2C and 2D) bilobed, endite with two short setae terminally; palp with three short and thick setae terminally. Both right and left maxillae linked basally by cuticular tympanum (Fig. 2J); subchelate, two-segmented, proximal segment stout, distal segment with sharp claw strongly curved inward. Maxilliped (Fig. 2K) subchelate, elongate and slender, two-segmented; corpus with three medial denticulate pads, middle of which bearing element; subchela smoothly curved inward, with denticulate pad distomedially and one claw and one small auxiliary spine terminally.

*Remarks*. *Brachiella malayensis* n. sp. closely resembles *B. magna* from Australia and *B. cybii* from India. These two known species were described only based on a single female. *Brachiella magna* was found on the gills of *S. commerson* in Australian waters [22]. Later, Pillai et al. [49] described *B. cybii*, found in the nasal cavity of *A. solandri* collected off Trivandrum, India.

*Brachiella malayensis* n. sp. can be distinguished from *B. magna* in: (1) body length (6.4 mm vs. 15 mm); (2) different proportions of the cephalosome, trunk, posterior processes and caudal rami (see Table 1); (3) the shape of dorsalmost tooth of the mandibular cutting edge (small vs. large); (4) the number of elements of the maxillulary endite (2 vs. 1); (5) lengths of setae on the maxillary palp (short vs. long); (6) group of denticles near the maxillipedal axilla (absent vs. present). *Brachiella cybii* was very briefly described on the basis of the habitus only by Pillai et al. [49]. However, *B. malayensis* n. sp. can be differentiated from it by the body size (6.4 mm vs. 20 mm) and different body proportions (Table 1).

The discovery of the male of the new species reminded us of similarities of males among the type species of *Brachiella*, *B. thynii*, and two other lernaeopodid genera *Charopinopsis* and *Eobrachiella*. The latter genera are synonymized with *Brachiella* in the present study. In fact, the male of *B. malayensis* n. sp. is very similar to those of *B. thynni* and *B. elegans* in having a long trunk, but is differentiated by the trunk longer than the cephalothorax (Table 2). The male is also distinguished in the shape of the maxillule, maxilla, and maxilliped from these two congeners (Table 2).

## Discussion

The family Lernaeopodidae has accommodated 46 genera and over 265 species to date, while 20 genera are monotypic [6]. According to Boxshall and Halsey [6], the taxonomy of this family “remains in urgent need of revision”. Although Wilson [64], Kabata [21–23, 25, 26], Kabata and Bowman [27], Castro Romero and Baeza Kuroki [9], and Piasecki et al. [46] intensively reviewed the taxonomy of the family, diagnoses of some genera are still ambiguous. This confusion is mainly due to profound modification in the body plan and superficial similarity of appendages, which sometimes makes the phylogenetic relationships obscure [25]. Kabata’s [25] vision of the evolutionary trends and phylogenetic relationships among lernaeopodids is suggestive as a landmark for re-definitions of these problematic taxa.

*Brachiella* can be well defined by the general body plans and long caudal rami of the females and the cephalic appendages of both sexes, especially the distinct synapomorphic state of the male maxillipeds, whereas it exhibits variability in morphology of other body parts of both sexes (Tables 1 and 2). In females, length ratios of cephalothorax to trunk, degrees of trunk expansion, and relative lengths of posterior processes to caudal rami are variable (Table 1). In males, the development of the trunk is highly variable (Table 2). Kabata [25] mentioned that the cephalosome, bulla, and trunk of lernaeopodid females are involved in feeding, attachment, and reproduction, respectively. The cephalosome and trunk of the males are also related to feeding and attachment to the mates and to reproduction, respectively. Therefore, it is likely that such interspecific variation depends on their own reproductive and adaptive strategies on different hosts.

The majority of the species related to the *Brachiella*-branch have been included in the genus *Parabrachiella*. Those with maxillary processes were relegated to *Thysanote* [45]. Only some of them were described based on both sexes. Therefore, the genus *Parabrachiella* constitutes a potential source of *Brachiella* species (as presently defined), after their males are discovered and properly described. Piasecki et al. [46] provided a checklist of valid species of *Parabrachiella* covering a total of 67 species. After 10 years, the list should be updated taking into consideration additional publications [14, 16, 31, 37, 48].

Recently, intraspecific variation of *Parabrachiella platensis* Montes, Castro-Romero et Martorelli, 2017 was found between specimens collected from nostrils and fins of *Mugil liza* Valenciennes. The species identification was confirmed by DNA barcoding using mt-DNA COI (similarity 99.8%) [37]. Minor morphological differences between these specimens were found especially in females: trunk length, shapes of cephalosome, anal slit, posterior truncal processes, labium and maxilla, and armature and elements of antennule, maxillule, and maxilliped [37]. On the other hand, *B. magna* and *B. malayensis* n. sp. found from the gills and nostrils of the same host *S. commerson*, respectively, also had a few differences as in the above-mentioned remarks. Thus, in the future, a comparison of the DNA sequences of *B. malayensis* n. sp. and *B. magna* might be useful to elucidate the validity of species if such variation also occurs between these two species. Although the distal tips (around the bulla) of the maxillae of *B. malayensis* n. sp. were accidentally lost during the handling procedure, if the bulla are located at the anteriormost part of the broken right maxilla (left shorter), such short maxillae seem to be adaptive in the narrower habitat, the nostril. The discovery of males of *B. magna* and *B. cybii* may confirm the present conclusion.

As Ho and Do [16] have precisely pointed out, males of *Brachiella* (as *Eobrachiella*) have an elongate maxilliped which suits embracing the cylindrical caudal ramus rather than pinching, implying avoidance of detachment from the mates on fast-swimming pelagic fish. In fact, hosts of *Brachiella* belong to Carangidae, Coryphaenidae, Peristediidae, Pomatomidae, Sciaenidae, and Scombridae, and Sphyraenidae which are mostly epipelagic [13] (Table 3). Ho and Do’s [16] hypothesis is supported by the present study. *In situ* sites of the males on the females were illustrated in some references. In *B. seriolae* and *B. quaternia*, a male of each species was illustrated to attach himself to the base of the ventroposterior process in Figures 10F, 13B, C and 14A, B of Ho and Do [16], respectively. On the other hand, a male adhered to the terminal portion of the ventroposterior process of a female of *B. thynni* (see [54]). Since paired copulatory pores are located between the ventroposterior processes, the males seem to prefer attachment to the ventroposterior processes rather than the dorsoposterior ones. Moreover, these different positions of the males on the female ventroposterior processes imply that they are capable of moving freely along the processes. The present study has focused more attention on the reproductive organs and behavior of lernaeopodids to improve understanding of the taxonomy and phylogeny of the family. In fact, free-living calanoid copepods exhibit highly variable configurations in the female reproductive systems (e.g., Barthélémy et al. [1]; Ohtsuka and Huys [41]), which was a key innovation to reconsider the phylogeny of the order.

**Table 3:** A comparison of *Brachiella* species in terms of their locality, host, and attachment site.

Species	Locality	Host	Family	Attachment site
*Brachiella malayensis* n. sp.	Terengganu, Malaysia	*Scomberomorus commerson* (Lacepède, 1800)	Scombridae	Nostrils
*Brachiella magna* Kabata, 1968	Queensland, Australia	*Scomberomorus commerson* (Lacepède, 1800)	Scombridae	Gills
*Brachiella cybii* Pillai, Prabha et Balaraman, 1982	Trivandrum, India	*Acanthocybium solandri* (Cuvier, 1832)	Scombridae	Nostrils
*Brachiella thynni* Cuvier, 1830	–	[A tuna, as suggested by the specific epithet]	Scombridae	Gills
	–	“*Scomber thynnus*” = *Thunnus thynnus* (Linnaeus, 1758)	Scombridae	Gills
	–	“Thon”	Scombridae	Gills
	Axelhulen, Denmark	“Albecorer” = *Thunnus alalunga*?	Scombridae	–
		“Baracottaer, Barracuder” = barracuda?	Sphyraenidae	
	Trieste, Italy	“Thynnusagtige Makrelfisk” = tuna-like mackerel fish	Scombridae	–
	Coasts of Belgium	*Sciaena umbra* Linnaeus, 1758		Gills
	Plymouth, England	“*Thynnus thynnus*” = *Thunnus thynnus* (Linnaeus, 1758)	Scombridae	Pectoral fin
	Mediterranean	“*Thynnus vulgaris*” = *Thunnus thynnus* (Linnaeus, 1758)	Scombridae	Gills
	Plymouth, England	“*Thynnus thynnus*” = *Thunnus thynnus* (Linnaeus, 1758)	Scombridae	
		“*Sciaena aquilla*” = *Argyrosomus regius* (Asso, 1801)	Sciaenidae	
As “*Thynnicola Ziegleri*”	Bakar, Croatia	“*Thynnus thynnus*” = *Thunnus thynnus* (Linnaeus, 1758)	Scombridae	Pectoral fin
	Polperro, Cornwall, England	“*Thynnus thynnus*” = *Thunnus thynnus* (Linnaeus, 1758)	Scombridae	–
	–	“*Orcynus thynnus*” = *Thunnus thynnus* (Linnaeus, 1758)	Scombridae	Gills
	Cornwall, England	“tunny fish”	Scombridae	Gills
	Bakar, Croatia	“*Thynnus thynnus*” = *Thunnus thynnus* (Linnaeus, 1758)	Scombridae	Gills
	Ceylon (Sri Lanka)	*Pomatomus saltatrix* (Linnaeus, 1766)	Pomatomidae	Axil of pectoral fin
	Gulf of Mexico			
	Off Owase, Japan	*Acanthocybium solandri* (Cuvier, 1832)	Scombridae	Around fins
	Hawaii	*Acanthocybium solandri* (Cuvier, 1832)	Scombridae	external surface
	British waters?	–	–	–
	Kerala, India [Other localities and hosts’ names were probably cited after other authors, based on all available records]	*Thunnus thynnus* (Linnaeus, 1758)	Scombridae	–
		“*Thunnus macropterus*” = *Thunnus albacares* (Bonnaterre, 1788)	Scombridae	
		*Acanthocybium solandri* (Cuvier, 1832)	Scombridae	
		*Chirocentrus dorab* (Forsskål, 1775)	Chirocentridae	
		“*Indocybium lineolatum*” *= Scomberomorus lineolatus* (Cuvier, 1829)	Scombridae	
		“*Orcynus thynnus*” = *Thunnus thynnus* (Linnaeus, 1758)	Scombridae	
		*Pomatomus saltatrix* (Linnaeus, 1766)	Pomatomidae	
		“*Sciaena aquilla*” = *Argyrosomus regius* (Asso, 1801)	Sciaenidae	
		*Sciaena umbra* Linnaeus, 1758	Sciaenidae	
		“*Sciaena rubra*” = *Sargocentron rubrum* (Forsskål, 1775)	Sciaenidae	
		“*Scomberomorus cavalla*” = *Scomberomorus maculatus* (Mitchill, 1815)	Scombridae	
	Yeosu, Korea	*Thunnus alalunga* (Bonnaterre, 1788)	Scombridae	Body surface
*Brachiella elegans* Richiardi, 1880	Italy	“*Lichia glauca*” = *Trachinotus ovatus* (Linnaeus, 1758)	Carangidae	Branchial arches
“*Brachiella* sp. (*elegans* Rich.?)”	Portoferraio, Tyrrhenian Sea	*Lichia amia* (Linnaeus, 1758)	Carangidae	Gill cavity
	Woods Hole, USA, Atlantic	*Seriola lalandi* Valenciennes, 1833	Carangidae	Gill cavity
“*Eobrachiella elegans*” ([64] specimens)	Woods Hole, USA, Atlantic	*Seriola lalandi* Valenciennes, 1833	Carangidae	Gill cavity
“*Charopinus quaternius* Wilson, 1935”	Dry Tortugas, Gulf of Mexico	*Peristedion gracile* Goode et Bean, 1896	Peristediidae	Gills
		*Coryphaena hippurus* Linnaeus, 1758	Coryphaenidae	Gills
As “*Brachiella coryphaenae* Pearse, 1952”	Gulf of Mexico	*Coryphaena hippurus* Linnaeus, 1758	Coryphaenidae	Gills and opercula
“*Charopinus quaternius*”	Grand Isle, IL, USA	“*Scomberomorus cavalla*” = *Scomberomorus maculatus* (Mitchill, 1815)	Scombridae	–
As “*Brachiella coryphaenae*”	Vizhington, India	*Coryphaena hippurus* Linnaeus, 1758	Coryphaenidae	Gill filaments
“*Charopinopsis quaternia*”	–	– (In reference to [65] records)	–	–
“*Charopinopsis quaternia*”	–	– (based on Wilson’s [65] specimens)	–	–
“*Charopinopsis quaternia*”	Oahu, Hawaii	*Coryphaena hippurus* Linnaeus, 1758	Coryphaenidae	Gill filaments
“*Charopinopsis quaternia*”	Daito Is., Japan	*Coryphaena hippurus* Linnaeus, 1758	Coryphaenidae	Gill
“*Charopinopsis quaternia*”	Key West, Gulf of Mexico	*Coryphaena hippurus* Linnaeus, 1758	Coryphaenidae	Gills
*Brachiella quaternia* new comb.				
*Brachiella seriolae* Yamaguti et Yamasu, 1960	Kojima Bay, Japan	*Seriola quinqueradiata* Temminck et Schlegel, 1845	Carangidae	Pectoral fins
“*Eobrachiella elegans* f. *seriolae*”	Kojima Bay, Japan	*Seriola quinqueradiata* Temminck et Schlegel, 1845	Carangidae	Pectoral fins

Species	Locality	Host	Family	Attachment site	Reference
*Brachiella malayensis* n. sp.	Terengganu, Malaysia	*Scomberomorus commerson* (Lacepède, 1800)	Scombridae	Nostrils	Present study
*Brachiella magna* Kabata, 1968	Queensland, Australia	*Scomberomorus commerson* (Lacepède, 1800)	Scombridae	Gills	[22]
*Brachiella cybii* Pillai, Prabha et Balaraman, 1982	Trivandrum, India	*Acanthocybium solandri* (Cuvier, 1832)	Scombridae	Nostrils	[49]
*Brachiella thynni* Cuvier, 1830	–	[A tuna, as suggested by the specific epithet]	Scombridae	Gills	[11]
	–	“*Scomber thynnus*” = *Thunnus thynnus* (Linnaeus, 1758)	Scombridae	Gills	[61]
	–	“Thon”	Scombridae	Gills	[36]
	Axelhulen, Denmark	“Albecorer” = *Thunnus alalunga*?	Scombridae	–	[56]
		“Baracottaer, Barracuder” = barracuda?	Sphyraenidae		
	Trieste, Italy	“Thynnusagtige Makrelfisk” = tuna-like mackerel fish	Scombridae	–	[56]
	Coast of Belgium	*Sciaena umbra* Linnaeus, 1758		Gills	[59]
	Plymouth, England	“*Thynnus thynnus*” = *Thunnus thynnus* (Linnaeus, 1758)	Scombridae	Pectoral fin	[2]
	Mediterranean	“*Thynnus vulgaris*” = *Thunnus thynnus* (Linnaeus, 1758)	Scombridae	Gills	[7]
	Plymouth, England	“*Thynnus thynnus*” = *Thunnus thynnus* (Linnaeus, 1758)	Scombridae		[3]
		“*Sciaena aquilla*” = *Argyrosomus regius* (Asso, 1801)	Sciaenidae		
As “*Thynnicola Ziegleri*”	Bakar, Croatia	“*Thynnus thynnus*” = *Thunnus thynnus* (Linnaeus, 1758)	Scombridae	At pectoral fin	[35]
	Polperro, Cornwall, England	“*Thynnus thynnus*” = *Thunnus thynnus* (Linnaeus, 1758)	Scombridae	–	[39]
	–	“*Orcynus thynnus*” = *Thunnus thynnus* (Linnaeus, 1758)	Scombridae	Gills	[53]
	Cornwall, England	“tunny fish”	Scombridae	Gills	[64]
	Bakar, Croatia	“*Thynnus thynnus*” = *Thunnus thynnus* (Linnaeus, 1758)	Scombridae	Gills	[64]
	Ceylon (Sri Lanka)				[29]
	Gulf of Mexico	*Pomatomus saltatrix* (Linnaeus, 1766)	Pomatomidae	Axil of pectoral fin	[4]
	Off Owase, Japan	*Acanthocybium solandri* (Cuvier, 1832)	Scombridae	Around fins	[54]
	Hawaii	*Acanthocybium solandri* (Cuvier, 1832)	Scombridae	External surface	[33]
	British waters?	–	–	–	[25]
	Kerala, India [other localities and host names were probably cited after other authors, based on all available records]	*Thunnus thynnus* (Linnaeus, 1758)	Scombridae	–	[48]
		“*Thunnus macropterus*” = *Thunnus albacares* (Bonnaterre, 1788)	Scombridae		
		*Acanthocybium solandri* (Cuvier, 1832)	Scombridae		
		*Chirocentrus dorab* (Forsskål, 1775)			
		“*Indocybium lineolatum*” *= Scomberomorus lineolatus* (Cuvier, 1829)			
		“*Orcynus thynnus*” = *Thunnus thynnus* (Linnaeus, 1758)	Scombridae		
		*Pomatomus saltatrix* (Linnaeus, 1766)	Pomatomidae		
		“*Sciaena aquilla*” = *Argyrosomus regius* (Asso, 1801)	Sciaenidae		
		*Sciaena umbra* Linnaeus, 1758	Sciaenidae		
		“*Sciaena rubra*” = *Sargocentron rubrum* (Forsskål, 1775)	Sciaenidae		
		“*Scomberomorus cavalla*” = *Scomberomorus maculatus* (Mitchill, 1815)	Scombridae		
	Yeosu, Korea	*Thunnus alalunga* (Bonnaterre, 1788)	Scombridae	Body surface	[60]
*Brachiella elegans* Richiardi, 1880	Italy	“*Lichia glauca*” = *Trachinotus ovatus* (Linnaeus, 1758)	Carangidae	Branchial arches	[51]
“*Brachiella* sp. (*elegans* Rich.?)”	Portoferraio, Tyrrhenian Sea	*Lichia amia* (Linnaeus, 1758)	Carangidae	Gill cavity	[8]
	Woods Hole, USA, Atlantic	*Seriola lalandi* Valenciennes, 1833	Carangidae	Gill cavity	[64]
“*Eobrachiella elegans*” ([65] specimens)	Woods Hole, USA, Atlantic	*Seriola lalandi* Valenciennes, 1833	Carangidae	Gill cavity	[16]
“*Charopinus quaternius* Wilson, 1935”	Dry Tortugas, Gulf of Mexico	*Peristedion gracile* Goode et Bean, 1896	Peristediidae	Gills	[65]
		*Coryphaena hippurus* Linnaeus, 1758	Coryphaenidae	Gills	
As “*Brachiella coryphaenae* Pearse, 1952”	Gulf of Mexico	*Coryphaena hippurus* Linnaeus, 1758	Coryphaenidae	Gills and opercula	[42]
“*Charopinus quaternius*”	Grand Isle, IL, USA	“*Scomberomorus cavalla*” = *Scomberomorus maculatus* (Mitchill, 1815)	Scombridae	–	[10]
As “*Brachiella coryphaenae*”	Vizhington, India	*Coryphaena hippurus* Linnaeus, 1758	Coryphaenidae	Gill filaments	[47]
“*Charopinopsis quaternia*”	–	– (In reference to [65] records)	–	–	[66]
“*Charopinopsis quaternia*”	–	– (based on Wilson’s [66] specimens	–	–	[21]
“*Charopinopsis quaternia*”	Oahu, Hawaii	*Coryphaena hippurus* Linnaeus, 1758	Coryphaenidae	Gill filaments	[33]
“*Charopinopsis quaternia*”	Daito Is., Japan	*Coryphaena hippurus* Linnaeus, 1758	Coryphaenidae	Gill	[28]
“*Charopinopsis quaternia*”	Key West, Gulf of Mexico	*Coryphaena hippurus* Linnaeus, 1758	Coryphaenidae	Gills	[16]
*Brachiella quaternia* new comb.					Present study
*Brachiella seriolae* Yamaguti et Yamasu, 1960	Kojima Bay, Japan	*Seriola quinqueradiata* Temminck et Schlegel, 1845	Carangidae	Pectoral fins	[67]
“*Eobrachiella elegans* f. *seriolae*”	Kojima Bay, Japan	*Seriola quinqueradiata* Temminck et Schlegel, 1845	Carangidae	Pectoral fins	[16]

Fish name validity verified after Fricke et al. [12]; –: no data provided.
